# Use of multilocus sequence typing to infer genetic diversity and population structure of *Lactobacillus plantarum* isolates from different sources

**DOI:** 10.1186/s12866-015-0584-4

**Published:** 2015-10-28

**Authors:** Haiyan Xu, Wenjun Liu, Wenyi Zhang, Jie Yu, Yuqin Song, Bilige Menhe, Heping Zhang, Zhihong Sun

**Affiliations:** Key Laboratory of Dairy Biotechnology and Engineering, Ministry of Education, Inner Mongolia Agricultural University, Hohhot, Inner Mongolia 010018 China

**Keywords:** *Lactobacillus plantarum*, Multilocus sequence typing, Housekeeping genes, Genetic diversity, Population structure

## Abstract

**Background:**

*Lactobacillus plantarum* is a lactic acid bacterium (LAB) of considerable industrial interest since it has an important role in the production of fermented food. In the present study, the genetic diversity and population structure within 186 *L. plantarum* isolates was determined based on a novel MLST scheme employing eight housekeeping genes. These isolates had originated from different sources and geographic regions: 179 isolates were from our own culture collection and originated from China and Mongolia and seven isolates were type or reference isolates from other collections.

**Results:**

The results showed that 179 isolates and seven reference isolates could be assigned to 73 different sequence types (STs), forming ten clonal complexes (CCs) and 23 singletons. There were 158 polymorphic sites detected in total, and the nucleotide diversity per site varied from 0.00401 in *clpX* to 0.03220 in *groEL*. The minimum spanning tree analyses suggested that the evolution of *L. plantarum* isolates have little relationship with ecological sources have similar nucleotide diversity. Phylogenetic trees and structure indicated that there were six lineages in the *L. plantarum* isolates used in our study. Split-decomposition and ClonalFrame analysis indicated that recombination had occurred throughout the population of *L. plantarum*, but it occurred at a low frequency in these eight loci.

**Conclusion:**

We deduced that *L. plantarum* isolates from the same ecological niches have similar genetic diversity and population structure. The MLST scheme presented in this study provides abundant sequence data for *L. plantarum* and enabled global comparisons of isolates associated with various environmental origins to be made. This will further advance our understanding of the microbial ecology of this industrially important LAB.

**Electronic supplementary material:**

The online version of this article (doi:10.1186/s12866-015-0584-4) contains supplementary material, which is available to authorized users.

## Background

*Lactobacillus plantarum*, is an ubiquitous species of lactic acid bacterium (LAB) that is found in a wide range of different ecological niches including vegetable, meat and dairy substrates, and the gastro-intestinal tract [1]. The diversity of niches occupied by *L. plantarum* is related to its capacity to ferment a broad range of sugars [2]. Isolates of *L. plantarum* have been widely used as starter cultures in the production of fermented vegetables and other crops [3–5], fermented dairy products such as cheese [6, 7], fermented meat products [8, 9], and as a probiotic for humans and animals [10].

*Lactobacillus plantarum* is closely related to the species *Lactobacillus fabifermentans*, *Lactobacillus paraplantarum*, *Lactobacillus pentosus* and *Lactobacillus xiangfangensis* and, within *L. plantarum*, a number of subspecies have been identified. These include *L. plantarum* subsp. *argentoratensis* and *L. plantarum* subsp. *plantarum* [11]. These closely related species and subspecies have similar nutritional requirements and, as such, they are difficult to separate using classical methods [12]. However, interspecific and intraspecific differentiation is an important preliminary step in the selection of starter cultures. Technological, probiotic, antimicrobial and sensorial attributes are strain-specific and so it is important to be able to distinguish between isolates with different properties [13]. For this reason, a great number of, molecular, techniques have been developed for the identification and typing of *L. plantarum* isolates. These include randomly amplified polymorphic DNA, pulsed-field gel electrophoresis, amplified fragment length polymorphism, repetitive element PCR and multilocus sequence typing (MLST) [13–17].

The MLST method was first described in 1998 [18] and since then has been used widely to study the population structure of important bacterial pathogens [19, 20]. Recently, research evidence has suggested that it could also be a powerful technique for typing LAB, allowing precise identification and easy comparison/exchange of results between different laboratories [21–23]. Previously, MLST has been used for the typing of *L. plantarum* isolates from fermented fruits and vegetables [16], and from silage, wine, pickled cabbage and cheese [13]. However, the number of isolates evaluated was rather limited, making any interpretation of the genetic heterogeneity of *L. plantarum* very difficult, and, potentially misleading*.* In the present study, a new MLST scheme was established and applied to a large number of *L. plantarum* isolates (179 plus seven reference and type isolates) from a wide range of geographic regions and different sources, to encompass the full genetic diversity and population structure of the species.

## Result

### Sequence types and clonal complexes

The partial sequences of the eight housekeeping genes (from 179 isolates and seven reference isolates of *L. plantarum*) ranged in size from 415 to 641 bp. A total of 73 unique STs were resolved, of which 44 corresponded to single isolates (Additional file 1: Table S1). ST47 contained the largest number of isolates, 19, which represented 10.2 % of all isolates evaluated (Additional file 1: Table S1). Furthermore, four of the reference isolates were assigned to STs (and CCs) with other isolates tested while the remaining three reference isolates were singletons in their own ST (Additional file 1: Table S1).

**Fig. 1:**
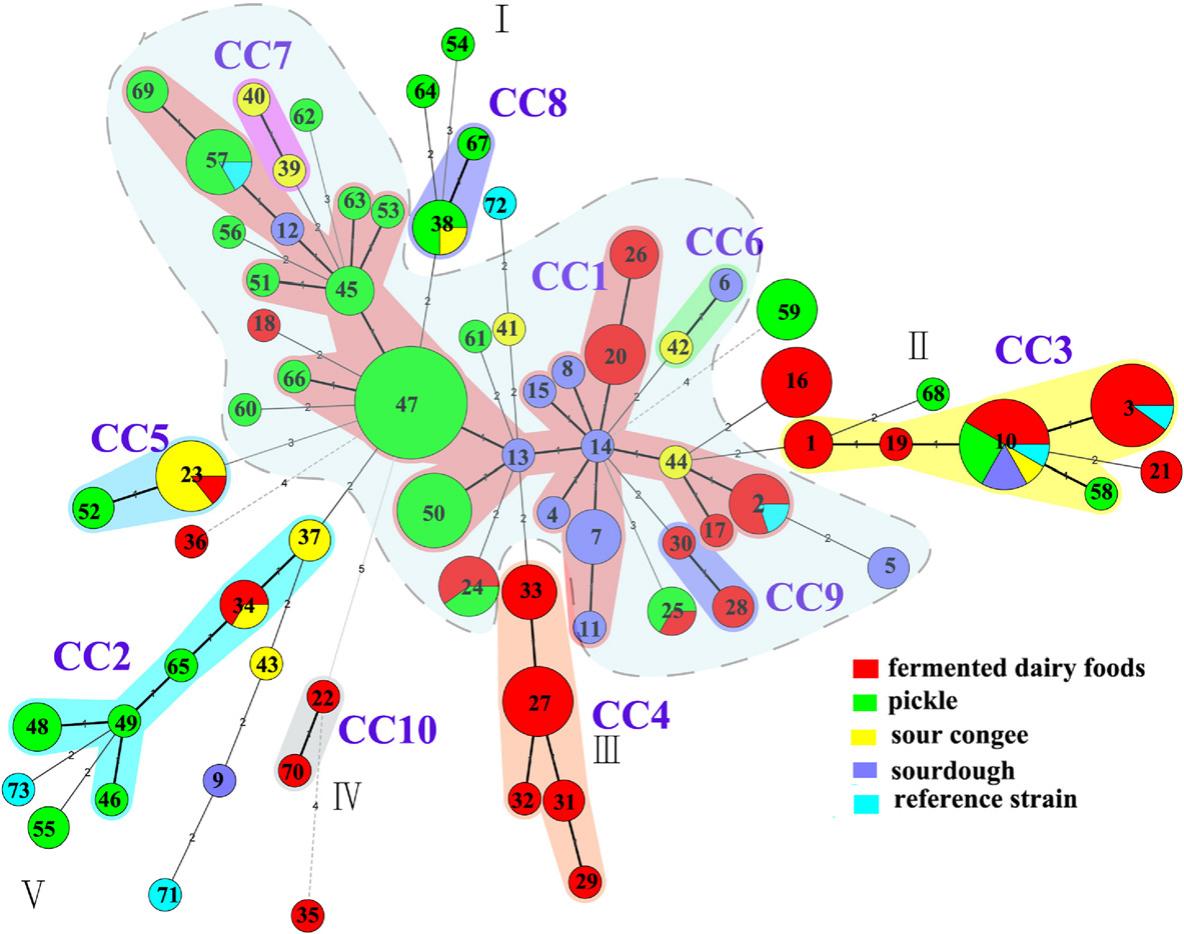
Minimum spanning trees analysis of the 186 *L. plantarum* isolates evaluated based on eight loci. Each circle corresponds to a sequence type (ST), and the circle size corresponds to the number of isolates sharing the same ST, the circle was coded by assigning the same colour to identical ecological source. The shaded zones between certain groups of circles indicate that these profiles belong to the same CC. Numerals connecting the circles indicate the number of allelic differences between the profiles. The strength of the link (bold, plain, or discontinuous) is correlated to the genetic similarity (number of common alleles) between profiles. Black lines connecting pairs of STs indicate that they share seven (thick lines), six (thin) or five alleles (dotted). No lines are present between pairs of STs that share less than four alleles

The 73 STs could be classified into ten CCs and 23 singletons using eBURST (Additional file 1: Table S1). The ten CCs contained 77.5 % of the isolates (Additional file 2: Figure S1). CC1 (22 STs) contained 70 isolates, ST14 was associated with seven single locus variants (SLVs), which was the predicted founder genotype [24]. CC2, CC3 and CC4 contained six (11 isolates), five (27 isolates) and five (15 isolates) STs, respectively. All other CCs contained fewer than five STs, each with a limited number of isolates. The links among CCs and singletons disclosed by eBURST profile were consistent with minimum spanning trees (Additional file 2: Figure S1 and Fig. 1).

### Genetic diversity

The nucleotide diversity within each of the eight housekeeping genes for all isolates was calculated and between seven (*pyrG*) and 15 (*murE*) alleles were found (Table 1). In total there were 158 polymorphic sites with between eight and 63 per gene (Table 1). The nucleotide diversity per site (π, the average number of nucleotide differences per site between two randomly-selected isolates) among the eight genes varied from 0.00401 (*clpX*) and 0.03220 (*groEL*). In addition, the number of sSNPs (synonymous single nucleotide polymorphism sites) was greater than the number of nSNPs (non-synonymous Single Nucleotide Polymorphism sites) except for *groEL*, which had 59 nSNPs and 6 sSNPs, the largest among all the genes. The DNA G + C% content of the different gene fragments ranged from 43.10 (*uvrC*) to 51.38 % (*murE*) and the proportion of concatenated sequences was 45.28 %; The *d*_*N*_/*d*_*S*_ ratios for the genes were between 0 (*recA*) and 0.2692 (*murC*) respectively, and were all lower than 1 (Table 1).

**Table 1:** Descriptive analysis of MLST data genetic variability at *L. plantarum* loci

Gene		No. of		π^a^/site	G + C content (mol%)			*d*_*N*_/*d*_*S*_^b^
	Length (bp)	Alleles	Polymorphic sites			sSNP	nSNP	
*clpX*	443	9	8	0.00401	45.77 %	7	1	0.0429
*groEL*	641	11	63	0.03220	43.93 %	6	59	0.0177
*murC*	462	11	10	0.00502	43.66 %	5	4	0.2692
*murE*	607	15	22	0.00855	51.38 %	16	6	0.0436
*pheS*	536	12	15	0.00693	46.09 %	10	5	0.0648
*pyrG*	415	7	9	0.00803	43.28 %	8	1	0.0255
*recA*	546	9	10	0.00549	45.00 %	10	0	0
*uvrC*	620	12	21	0.01166	43.10 %	18	3	0.0279
concatenated	4251	80	158	0.00464	45.28 %	80	79	0.0401

^a^Mean pairwise nucleotide difference per site

^b^ *d*_N_/*d*_S_ represents the ratio of nonsynonymous to synonymous substitutions

### Phylogenetic relationships analysis

Analysis of the allelic profiles of all 186 isolates generated a minimum spanning tree that provided an intuitive view of the phylogenetic relationships between STs, singletons, CCs and lineages (Fig. 1). One dominant group (in blue shading) and some small clades (Clades I-V) were discerned. The dominant group included CC1, CC6, CC7, CC9 and some singletons, and primarily contained isolates from Sichuan pickle (to the left of the group in Fig. 1) or sourdough and a small number of isolates were from fermented dairy foods (to the right of the group in Fig. 1). Clade I contained seven isolates from four STs (ST38, ST54, ST64 and ST67) of which six isolates came from pickles. Clade II contained isolates with the greatest diversity of ecological sources, although isolates in ST3 all originated from fermented dairy foods including reference isolate CGMCC6312 [25]. Clade III was composed of five STs, each from a different region, but all containing isolates from fermented dairy foods. Similarly, isolates in Clade IV were all from fermented dairy foods. Most of the STs in Clade V were isolated from pickles.

### Population structure

**Fig. 2:**
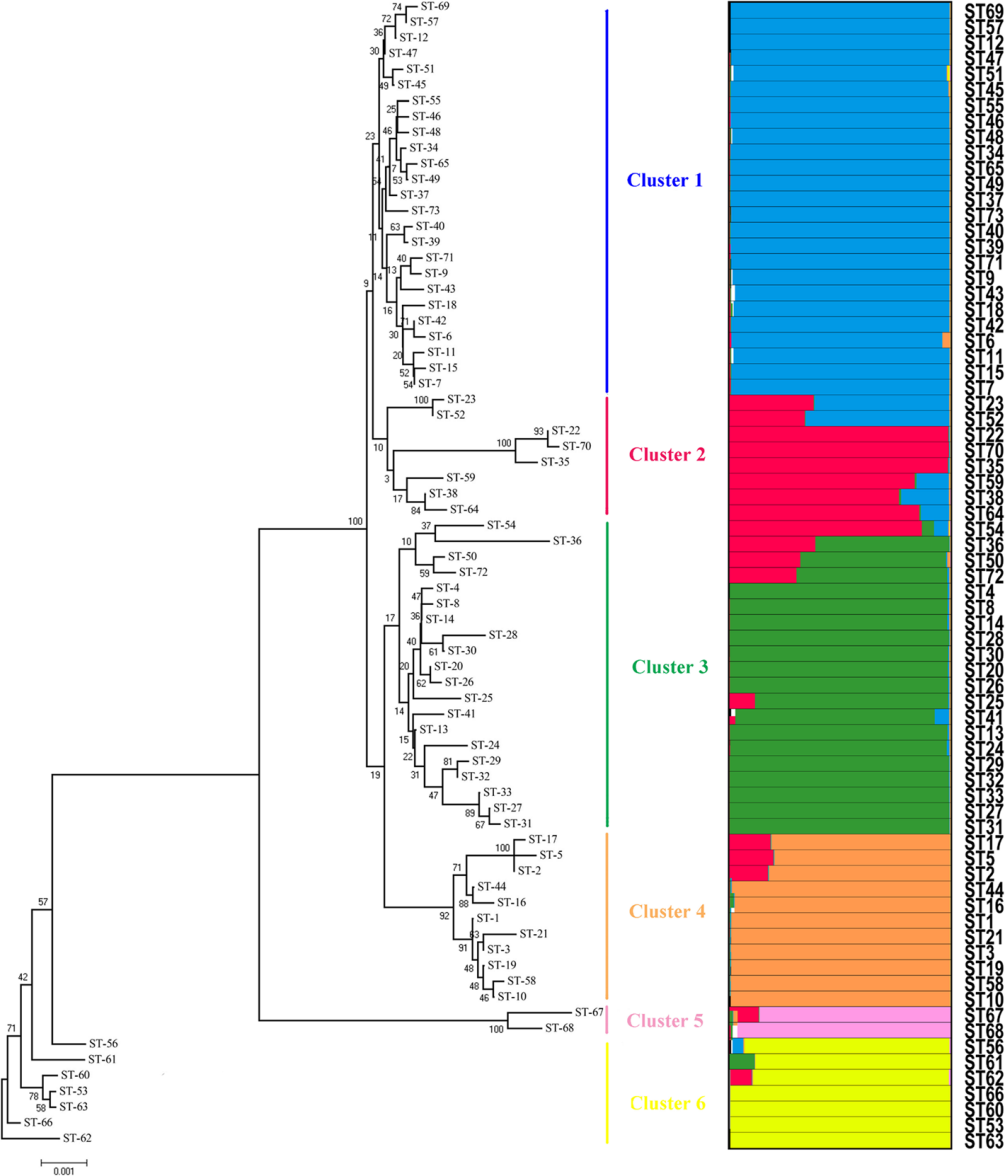
Neighbour-Joining tree and Ancestry of 73 *L. plantarum* STs. The left of the figure: Neighbour-Joining tree constructed from 73 concatenated nucleotide sequences of eight genes used in MLST. Bootstrap values are indicated for all branches. The right of the figure: The sources of ancestry of each unique ST from six ancestral populations by STRUCTURE. Each ST is represented by a single line with the ST designation at the top consisting of colour stacked bars that indicate the proportion of ancestry from each of six populations (blue, red, bottle green, orange, pink and yellow)

The concatenated sequences size of the eight housekeeping genes of 186 *L. plantarum* isolates was 4251 bp. A Neighbour-joining tree of the concatenated sequences of 73 STs was in almost complete agreement with the STRUCTURE which revealed six major ancestral populations, or clusters (Cluster 1–6) (Fig. 2). From the Neighbour-joining tree Cluster 1 (blue) comprised 25 STs and 59 isolates, of which 94.9 % came from non-dairy sources, and included reference isolates CGMCC0847 and CGMCC12167 and the type isolate ATCC 14917^T^. Cluster 2 (red) contained eight STs, and the distances amongst ST22, ST35 and ST70 from dairy sources were closer than amongst the other STs from non-dairy sources. The 20 STs in Cluster 3 (bottle green) included isolates from multiple sources, although 62.0 % of 50 isolates were from fermented dairy foods from different areas, including reference isolate NCIMB8826. Cluster 4 (orange) contained 44 isolates in 11 STs; 77.3 % of these isolates were from fermented dairy food and included reference isolates CGMCC12986 and CGMCC6312. Cluster 5 (pink) contained only two STs, both with isolates from pickle. Cluster 6 (yellow) contained seven STs, all with isolates from pickles.

The STRUCTURE showed six distinct clusters representing six ancestral populations (shown in six different colours) within 73 STs (each vertical line represented one ST). Isolates in Cluster 1 were isolated from non-fermented dairy products from different regions. However, some isolates from the same food origin were also divided between Clusters; isolates from pickle in Sichuan were divided between Clusters 5 and 6. Cluster 2, 3 and 4 were very diverse in origin but we identified the same ancestral sources with particular. The ancestry of each isolate was estimated as the summed probability of derivation from each cluster over all polymorphic nucleotides (shown at the right of Fig. 2). There were little admixture of ancestral sources in these clusters, with the exception of ST2, ST5, ST17, ST23, ST36, ST38, ST50, ST52 and ST72 (a percentage of assignment to a cluster lower than 85 % is considered as threshold for admixture), which seemed to have acted frequently both as donors and as recipients of recombination exchanges.

### Recombination analysis

Analysis of all isolates indicated the index of association (*I*_A_) of −0.0382 (*P* = 0.66) and standardized index of association (*I*_A_^   S^) of −0.0055 (*P* = 0.57), suggesting no significant linkage disequilibrium and indicating a tendency for free recombination between the alleles.

**Fig. 3:**
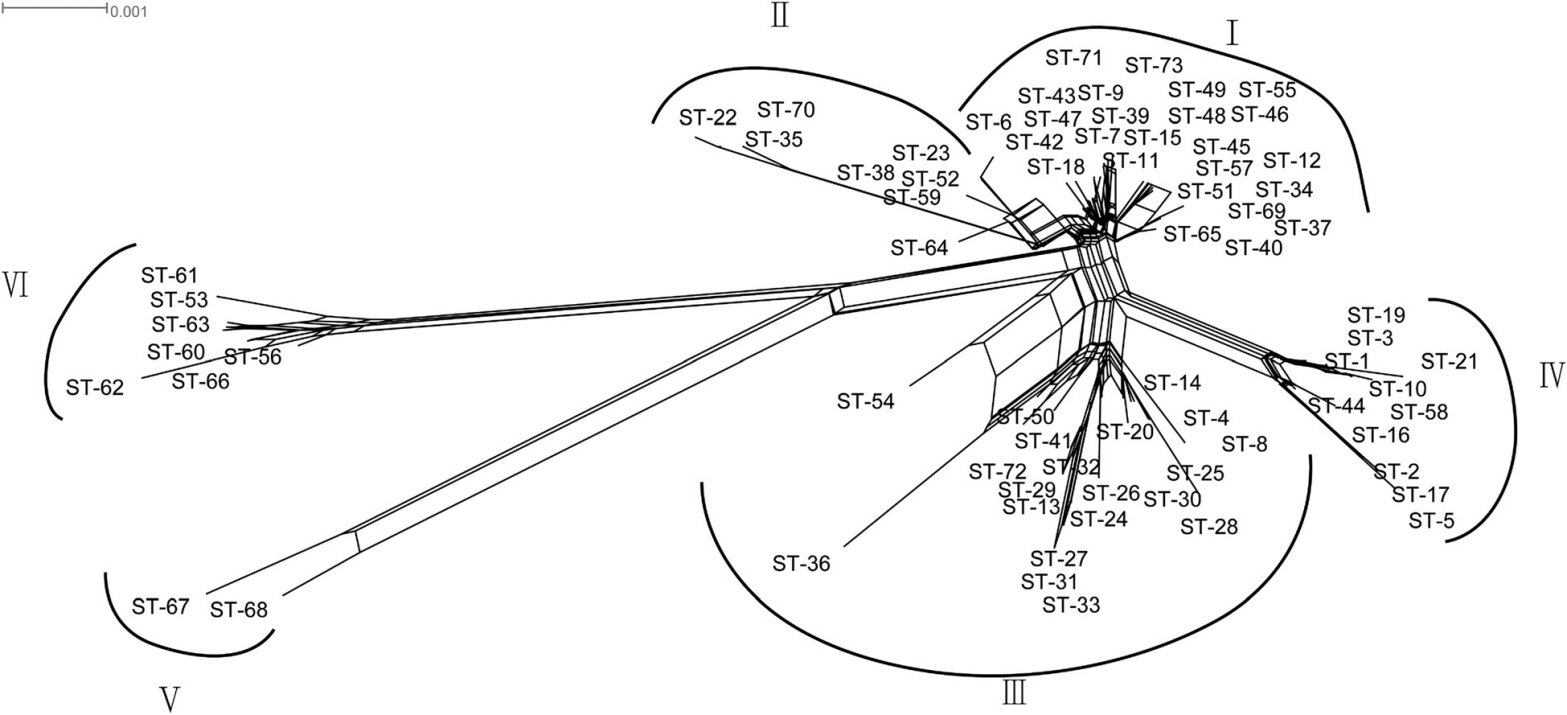
Split decomposition analysis based on all eight genes used in MLST

Split decomposition analysis of the concatenated *clpX, groEL, murC, murE, phesS, pyrG, recA* and *uvrC* gene sequence fragments displayed in a split decomposition tree (Fig. 3) showed a network-like structure with multiple parallel paths. There were six distinct clusters in the split decomposition tree, which was consistent with the phylogeny observed previously in the neighbour-joining tree and from STRUCTURE analysis.

**Fig. 4:**
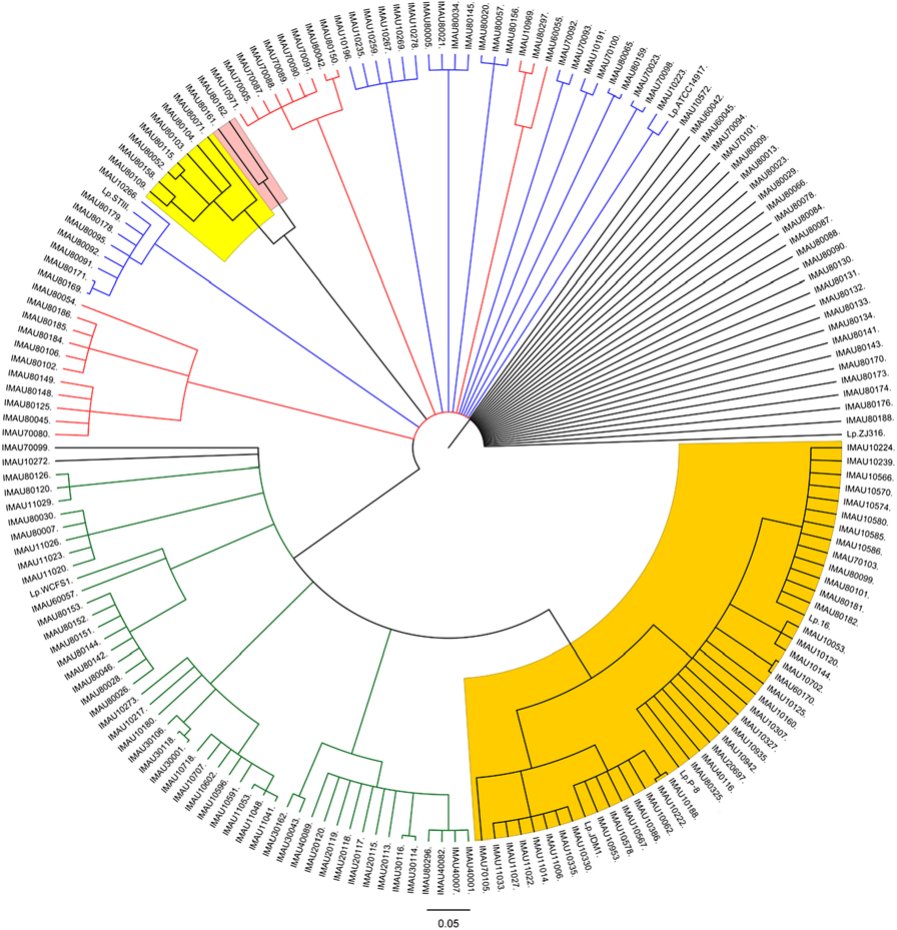
Clonal genealogy inferred from ClonalFrame analysis of our data using a 50 % majority-rule consensus tree. The clades from the ClonalFrame analysis correspond to the shadowed populations identified in Fig. 2 and have therefore been coloured with the same colours as in Fig. 2

ClonalFrame analysis estimated that recombination happened less frequently than mutation (ρ/θ = 0.18; Cl 95: [95 % credibility interval]: 0.07–0.33) and was less effective in introducing polymorphisms (γ/m = 0.64; Cl 95: 0.31–1.03). Clonal genealogy inferred from analysis of the data by ClonalFrame showed six clusters (Fig. 4). Clusters 4, 5 and 6 were the same clusters as identified by STRUCTURE (Fig. 2). The remaining three clusters found by STRUCTURE contain the remaining isolates in Fig. 3, but do not represent the different clusters in Fig. 4, Clusters 1, 2 and 3 from STRUCTURE analysis, were divided into smaller groups by ClonalFrame.

## Discussion

*Lactobacillus plantarum* is a LAB of considerable industrial interest, which plays a key role in the production of fermented food. Nevertheless the genetic diversity and population structure of this microorganism from different ecological origins had not been fully explored until this study. Here we used a new MLST scheme to examine isolates from a diversity of ecological sources. The new MLST scheme relied on sequencing of 400–650 bp fragments from eight housekeeping genes, which was a method previously proved to be useful for elucidating the phylogenetic relationships and evolution of fungal isolates [26]. However, an independent MLST scheme had been described [13] in which 16 isolates were tested for seven housekeeping gene fragments. Compared to the MLST schemes, an average of ten distinguishable alleles per locus among the 73 STs were identified, which is more than the average of 5 alleles observed from 16 *Lactobacillus plantarum* isolates. Our MLST scheme has stronger typing discrimination for *Lactobacillus plantarum*.

By comparison, the π values obtained for *L. plantarum* isolates in our study were higher than *L. delbrueckii* ranged from 0.0051 to 0.0096 [27] and for *L. plantarum* ranged from 0.0004 to 0.0072 [13], indicating a higher degree of intragenic nucleotide polymorphism in our isolates [13]. The sequences of eight housekeeping genes examined by MLST had mol% G + C contents that were similar to the genome of *L. plantarum* ATCC14917^T^ [28]. This suggests that these genes had been present in *L. plantarum* for a long period of time and were relatively conserved. Overall the *d*_*N*_*/d*_*S*_ ratios of the selected genes were lower than 1, which is typical of housekeeping genes and desirable in MLST schemes [29].

We investigated the evolutionary relationships amongst our isolates of *L. plantarum* by eBURST, based on alleles, and constructed a minimal spanning tree (Additional file 2: Figure S1 and Fig. 1). According to the minimal spanning tree, the dominant groups of isolates in blue shading and Clade I and Clade V, were associated with plant origins with most isolates coming from pickles, sourdough and sour congee. Notably in this dominant group, the reference isolate CGMCC0847 from kimchi [30] belonged to ST57 with other isolates from pickle; likewise, reference isolate CGMCC12986, a commercial lactic acid bacterium [31], was assigned to ST2 with other isolates from fermented dairy foods. Isolates in Clades III and IV, were all isolated from dairy sources, suggesting that *L. plantarum* isolates from identical or similar environmental sources were closely related. Curiously, the type isolate of *L. plantarum*, ATCC 14917 ^T^ from pickled cabbage shared six alleles with ST9 which was from sourdough [1], so the relationship between them was also close. Similarly, the reference isolate CGMCC12167 from a faecal sample was a two loci variant of ST49 isolated from pickle (Fig. 1). Furthermore, the reference isolates CGMCC12986 and CGMCC6312 were assigned to the same STs, with other isolates, and were isolated from identical sources. Therefore we can conclude that isolates from the same environmental sources have similar nucleotide diversity, maybe it’s just because different kinds of home-made fermented foods have an effect on the genetic evolution of *L. plantarum*, which existed in these traditional foods for a long time based on the special production process of these home-made foods. Our findings were different from de las Rivas, where there was little relationship with the sources of the isolates [13]. De las Rivas described most of the types were represented by a single strain, only strains isolated from the same source, such as strains CECT 223 and 224, and strains RM71 and 72 shared the same ST, but our results corroborated 39.7 % of the isolates shared STs. In addition, the type isolate of *L. plantarum* ATCC 14917 ^T^ and reference isolate NCIMB8826 represented a single ST in both schemes, respectively, maybe because our isolates were isolated from some special fermented foods. Similarly, the report on *L. sanfranciscensis* confirmed manufacturing environment was the predominant factor influencing the dominance of an isolate on the evolution of lactobacilli population [32]. Cai also proved that *L. casei* was adaptive evolution towards specific niches [33].

Results from the Neighbour-Joining tree corresponded well to the results from STRUCTURE analysis with respect to the identification of Clusters. STRUCTURE analysis recognized six ancestral sources of polymorphism within the *L. plantarum* isolates evaluated and these six groups had relationships with different environmental source. In addition, most STs were single ancestral source to contribute more than 85 %. However, within the entire dataset, some STs (16.1 % of all isolates) contained significant ancestry from multiple sources, where extensive recombination has yielded a highly heterogeneous set of isolates with multiple sources of ancestry (Fig. 2). This confirms previous studies demonstrating that the recombination plays a role in creating genetic heterogeneity in *L. plantarum* [13].

It is now clear that recombination plays a driving role in the evolution of bacteria [34], but little was known about the details of the recombination process within *L. plantarum*. In our study, multilocus linkage disequilibrium was analyzed using *I*_*A*_ and *I*_*A*_^   S^ for the recombination measurement of *L. plantarum*. The values of *I*_*A*_ and *I*_*A*_^   S^ were close to zero, which confirmed the importance of recombination in the distribution of alleles. Consistently, the split decomposition analysis of concatenated sequences displayed a very complex interconnecting network structure. Therefore, our data suggest that recombination has contributed to the evolution of *L. plantarum*, further confirmed by limited and frequent gene flow across clusters. These events may have facilitated rapid adaptation of *L. plantarum* to different environments. This is in agreement with previous reports that recombination is an important source of diversity in LAB [16].

Likewise, the ClonalFrame analysis confirmed that homologous recombination contributed to the evolution of *L. plantarum* to some degree. Based on the combined evidence from STRUCTURE and ClonalFrame analyses, Cluster 4, 5 and 6 can confidently be called lineages of *L. plantarum*. Two of these three lineages (Cluster 5 and 6) were closely related and they were all isolated from pickle. Interestingly, some clades in the ClonalFrame analysis were defined as groups of isolates that mainly had the same ancestry as in the Structure analysis, indicating that recombination happened predominantly between related isolates during the evolution of *L. plantarum* [34, 35]. Moreover, it remains likely that other small clades and single isolates exist and might be identified if a larger number of isolates were evaluated.

## Methods

### Isolates, media and culture conditions

Isolates evaluated were all from the Lactic Acid Bacteria Culture Collection of the Inner Mongolia Agriculture University, China (IMAU) with the exception of the reference and type isolates that were from the American Type Culture Collection (ATCC: isolate ATCC14917^T^), the China General Microbiological Culture Collection Center (CGMCC: isolates CGMCC 0847, 6312, 12167 and 12986), and the National Collection of Industrial and Marine Bacteria (NCIMB: isolates NCIMB 8826 and 41875) (Additional file 1: Table S2, Table S3). The IMAU isolates were originally isolated from home-made pickle, sour congee, sourdough and fermented dairy foods (cows’ milk, goats’ milk and mares’ milk) sampled in the Sichuan, Qinghai and Xinjiang provinces of China, the Inner Mongolia and Tibet autonomous regions of China, and the Dornogovi province of Mongolia. 187 *L. plantarum* strains were isolated from 187 different fermented food samples, which had been in mature stage. These home-made fermented foods are manufactured by natural fermentation with natural starter cultures (a stable microorganism formed during fermented process) from old fermented food, without the use of any commercial starter cultures (isolated and cultured by companies or departments to sell). In this way, *L. plantarum* was kept and handed down through the generations along with traditional home-made food recipes. All *L. plantarum* isolates had been achieved by 16S rRNA sequences [36–42] (Additional file 1: Table S2). Reference isolates were from dairy products, pickles, saliva and faecal samples from China, USA and UK (Additional file 1: Table S3). Each isolate was grown at 37 °C over night in 5.0 ml de Man Rogosa Sharpe broth (MRS; OXOID, CM0359B, Germany).

### DNA extraction and PCR amplification

Genomic DNA was extracted from each isolate using the methods of Yu [40] . DNA extraction the cells were pelleted by centrifugation at 8000 × g and resuspended in TE. Briefly, 1 mL of sample was frozen with liquid N_2_ for 5 min and thawed in a water bath at 65 °C. Then 600 μL of 400 mmol L^−1^NaOH and 300 μL of 400 g L^−1^ trisodium citrate dihydrate were added. The mixed sample was centrifuged at 8 000 × g for 5 min. The supernatant fluid was discarded. Afterwards, the sediment was dissolved in sterile ultrapure water. Then 50 μL of 100 g L^−1^ sodium dodecyl sulfate and 10 μL of 10 mg mL^−1^ proteinase K solution were added and the mixture was incubated at 55 °C for 1 h. After incubation, 10 μL of 5 mol L^−1^ NaCl and 100 μL of cetyltriethyl ammnonium bromide/NaCl (1:9 w/w) were added and the mixture was further incubated at 65 °C for 10 min. Subsequently, the mixture was extracted with 1 volume of phenol/chloroform/isoamyl alcohol (25:24:1 v/v/v) and chloroform/isoamyl alcohol (24:1 v/v) twice. Finally, DNA was precipitated by adding 0.1 volume of 3 mol L^−1^ sodium acetate to the water phase followed by 1 volume of ice-cold isopropyl alcohol. The DNA was collected, washed and dissolved in 50 μL of sterile ultrapure water. Purified DNA was diluted to a final concentration of 100 ng/μL for evaluation. For each isolate, genomic DNA was used as a template for PCR amplification of MLST loci using an automatic thermal cycler (PTC-200, MJ Research, Waltham, MA). Thermal cycling conditions were: 94 °C for 5 min; 30 cycles of 94 °C for 1 min, the appropriate temperature for the gene of interest for 1 min (Table 2), and 72 °C for 2 min; a final elongation step of 72 °C for 10 min. Each PCR reaction was performed in a volume of 50 μL containing 150 ng of genomic DNA, 8 mM of each dNTP, 10 pmol of each primer and 2.5 U *Taq* polymerase in 1× PCR buffer (with Mg^2+^). PCR products were electrophoresed in a 1.2 % agarose gel. The PCR products were sequenced by the Shanghai Majorbio Bio-pharm Technology Corporation. The same primers were used for PCR and sequencing of both DNA strands.

**Table 2:** The information of primers and housekeeping genes for MLST of *L. plantarum*

Gene	Template Length (bp)	Gene function	PCR primer	Sequences (5’-3’)	Annealing Temperature
*pheS*	717	Phenylalanyl-tRNA synthase alpha subunit	*pheS* _primerF	CCGTGAAGAACTGGAACA	49 °C
			*pheS* _primerR	CCTAACCCAAAGGCAAAA	
*pyrG*	668	CTP synthase	*pyrG*_primerF	AGTGATTTAGGTTCCGACAA	52 °C
			*pyrG*_primerR	TGCATTCCCAAGCAGATA	
*uvrC*	763	UvrABC system protein C	*uvrC* _primerF	GATCATTTATGTGGGTAAGGC	53 °C
			*uvrC* _primerR	TGACACTACTGGGAACAAGC	
*recA*	689	Recombinase A	*recA*_primerF	TTTTAGTTGTTGACTCGGTGGC	58 °C
			*recA*_primerR	TTCCGCTGGTGTCGCTTT	
*clpX*	563	ATP-dependent Clp protease subunit X	*clpX*_primerF	ATCGCCAAGAAGAGTGAA	52 °C
			*clpX*_primerR	ATAATCGAGCGTAGACCC	
*murC*	641	UDP-N-acetyl muramate-alanine ligase	*murC*_primerF	TATCGCTCCCACCAGTTA	51 °C
			*murC*_primerR	CGGCCAAGATTTCCTTAT	
*groEL*	737	60 kDa chaperonin	*groEL*_primerF	CGGCTACTTATCACAATACA	58 °C
			*groEL*_primerR	GCCTTCTAAACCAGCATT	
*murE*	750	UDP-N-acetylmuramyl tripeptide synthase	*murE*_primerF	ACTAATAAGGTCGCTGTTCTG	55 °C
			*murE*_primerR	TTTAGCGGCTTCTTCACT	

### MLST gene selection

Genes were selected on the basis of their location on the chromosome (preferably evenly separated across the entire genome), the functions of the encoded proteins (preferably conserved and well characterized) and their presence in all isolates as a single copy [43]. Eight housekeeping genes (*clpX, groEL, murC, murE, pheS, pyrG, recA* and *uvrC*) were selected for MLST analysis of *L. plantarum* isolates. These housekeeping genes were conserved sequence, their wide distribution across the chromosome, and their mutually unlinked in location. The *clpX* encodes ATP-dependent protease ATP-binding subunit *ClpX*, *groEL* encodes chaperonin GroEL, *murC* encodes UDP-N-acetylmuramyl tripeptide synthase, *murE* encodes UDP-Nacetylmuramoylalanyl-D-glutamate-2, 6-diaminopimelate ligase, *pheS* encodes phenylalanyl-tRNA synthase alpha subunit, *pyrG* encodes CTP synthetase, *recA* encodes recombinase A, *uvrC* encodes excinuclease ABC subunit C. The genes *pyr*G, *groEL* and *recA* had been used in a previous study on *Lactobacillus delbrueckii* [27], and *pheS* in a study on *Lactococcus lactis* [44]. The genes *clpX, murC, murE* and *uvrC* were known to have single nucleotide polymorphisms (SNPs) in one of the reference *L. plantarum* isolates, CGMCC6312 (Genbank number: CP005942). Primers for these genes were designed using the Primer Premier 5.0 programme (Premier Biosoft International) based on the complete genome of *L. plantarum* isolate CGMCC6312 (Table 2). For subsequent comparison we downloaded from Genbank the sequences of these eight genes in the reference and type isolates of *L. plantarum* that we used in this study (Additional file 1: Table S3).

### MLST data analysis

Sequence alignment, trim, analysis and the identification of polymorphic sites were performed using the MEGA 6.0 software package [45]. BioNumerics Software (version 6.0, Applied-Maths, SintMaartens-Latem, Belgium) was used for sequence assembly, definition of alleles and the construction of a minimum spanning tree. For each of the eight genes used in MLST, unique nucleotide sequences defined an allele. Unique allelic profiles for isolates consisted of unique combination of alleles (one from each gene), and were used to unambiguously define the sequence type (ST) of that isolate; several isolates could belong to the same ST if they shared the same allelic profile. Groups of isolates with closely related STs were grouped within clonal complexes(CCs) using eBURST [46] (http://eburst.mlst.net/).

The START version 2.0 programme [47] was used to calculate the number of polymorphic sites, the mean G + C% content of the DNA and the *d*_*N*_*/d*_*S*_ ratios (where *d*_*S*_ is the number of synonymous substitutions per synonymous site, and *d*_*N*_ is the number of non-synonymous substitutions per non-synonymous site). Using this software we also evaluated intragenic recombination within the eight MLST genes. Multilocus linkage disequilibrium between alleles was measured using the index of association (*I*_A_) and standardized index of association (*I*_A_^   S^) [48]. These statistics are expected to be zero when the alleles are in linkage equilibrium, which also indicates that frequent recombination events exist. We used DnaSP version 5.0 to calculate the nucleotide diversity (π) per site and polymorphic sites [49].

The sequences of the eight housekeeping genes of 186 *L. plantarum* isolates were concatenated. Phylogenetic trees based on concatenated sequences were constructed using the Neighbour-Joining method, with a Kimura two-parameter distance model in the MEGA 6.0 software package. The Bayesian analysis software STRUCTURE with an associated linkage model was used to identify the ancestral populations of each isolate [50]. We use a Bayesian clustering approach to assume a model in which there are K populations, each of which is characterized by a set of allele frequencies at each locus based on Markov Chain Monte Carlo (MCMC) iterations [51, 52]. Three independent runs were performed and each value for K, the number of ancestral populations, ranged from 2 to 10. Each run had 300,000 MCMC iterations, of which the first 200,000 iterations were discarded as burn-in. The optimal value was found to be K = 6 by comparing runs using the same values of K from 2 to 10.

We applied Splits Tree 4.0 [53, 54] and ClonalFrame [35] to assess recombination and mutation frequencies. The split decomposition method was performed based on the concatenated sequences of all alleles of the eight genes. To do ClonalFrame, a total of 100,000 MCMC iterations and three runs were performed, of which the first half was discarded as burn-in. To evaluate the relative impact of mutation rate (θ) and recombination rate (r) on the whole population of STs, r/θ (relative frequency of occurrence of recombination and mutation) and r/m (relative impact of recombination and mutation in the diversification of the lineages) were calculated.

### Nucleotide sequence accession numbers

Allele sequences for the eight genes used in MLST were deposited in the GenBank database under accession numbers from KP033527 to KP034958.

## Conclusion

We developed an MLST scheme for *L. plantarum* using 186 strains isolated from a range of different sources, and evaluated some basic parameters of its population biology. Our results indicate a low degree of recombination during the evolution of *L. plantarum*. Remarkably, the sources of isolates were possibly correlated with their diversity and population structure. In future studies we hope to further clarify these results by using more isolates from different sources.

### Availability of supporting data

The sequences data sets supporting the results of this article are available in the GenBank repository of National Center for Biotechnology Information (http://www.ncbi.nlm.nih.gov/nuccore/), and accession numbers were from KP033527 to KP034958.

## Additional files

Additional file 1: Table S1. Allelic profiles of analyzed *L. plantarum* strains. **Table S2.** Bacterial isolates used in this study. **Table S3**. Information of type strains and reference strains. (DOCX 29 kb) Additional file 2: Figure S1. Clonal genealogy inferred from ClonalFrame analysis of our data using a 50 % majority-rule consensus tree from three independent runs. The clades from the ClonalFrame analysis correspond to the shadowed populations identified in Fig. 2 and have therefore been coloured with the same colors as in Fig. 2. (DOC 90 kb)

## Abbreviations

MLST: Multilocus sequence typing
*L. plantarum*: *Lactobacillus plantarum*
LAB: Lactic acid bacterium
STs: Sequence types
CCs: Clonal complexes
sSNP: Synonymous single nucleotide polymorphism sites
nSNP: Non-synonymous single nucleotide polymorphism sit
I_A_: Index of association
I_A_^S^: Standardized index of association
*L. delbrueckii*: *Lactobacillus delbrueckii*
*L. sanfranciscensis*: *Lactobacillus sanfranciscensis*
*L. casei*: *Lactobacillus casei*
IMAU: Inner Mongolia Agriculture University
ATCC: American Type Culture Collection
CGMCC: China General Microbiological Culture Collection Center
NCIMB: National Collection of Industrial and Marine Bacteria
d_S_: Synonymous substitutions
d_N_: Non-synonymous substitutions
MCMC: Markov Chain Monte Carlo
