# Supplementary material for: Use of multilocus sequence typing to infer genetic diversity and population structure of Lactobacillus plantarum isolates from different sources
Source: BMC Microbiol. 2015 Oct 28;15:241. doi: 10.1186/s12866-015-0584-4 (PMC4625847; doi:10.1186/s12866-015-0584-4)
Supplement: Additional file 1: Table S1. — Allelic profiles of analyzed L. plantarum strains. Table S2. Bacterial isolates used in this study. Table S3. Information of type strains and reference strains. (DOCX 29 kb) [file 12866_2015_584_MOESM1_ESM.docx]

**Additional file 1**

**Table S1.** Allelic profiles of analyzed *L. plantarum* strains

| ST | *clpX* | *groEL* | *murC* | *murE* | *pheS* | *pyrG* | *recA* | *uvrC* | Group^*^ | Region (No.of isolates) | Source (No.of isolates) |
| --- | --- | --- | --- | --- | --- | --- | --- | --- | --- | --- | --- |
| 1 | 1 | 1 | 1 | 1 | 1 | 1 | 2 | 3 | CC3 | Inner Mongolia (3) | Fermented dairy foods (3), |
| 2 | 1 | 1 | 1 | 10 | 1 | 2 | 1 | 3 | CC1 | Inner Mongolia (4),  Reference strain (1) | Fermented dairy foods (4),  Culture starter (1) |
| 3 | 1 | 1 | 1 | 1 | 2 | 1 | 2 | 3 | CC3 | Inner Mongolia (6), Qinghai (1), Siichuan (1), China; Dornogovi, Mongolia (1), Reference strain (1) | Fermented dairy foods (10) |
| 4 | 1 | 7 | 1 | 1 | 1 | 2 | 1 | 1 | CC1 | Inner Mongolia (1) | Sourdough (1) |
| 5 | 1 | 1 | 1 | 10 | 1 | 2 | 5 | 11 | S1 | Inner Mongolia (2) | Sourdough (2) |
| 6 | 1 | 1 | 6 | 1 | 2 | 2 | 1 | 2 | CC6 | Inner Mongolia (1) | Sourdough (1) |
| 7 | 1 | 1 | 1 | 1 | 1 | 2 | 1 | 5 | CC1 | Inner Mongolia (4) | Sourdough (4) |
| 8 | 7 | 1 | 1 | 1 | 1 | 2 | 1 | 1 | CC1 | Inner Mongolia (1) | Sourdough (1) |
| 9 | 6 | 1 | 1 | 1 | 3 | 2 | 2 | 2 | S2 | Inner Mongolia (1) | Sourdough (1) |
| 10 | 1 | 1 | 1 | 1 | 2 | 1 | 2 | 4 | CC3 | Inner Mongolia (8), Sichuan (3), Reference strain (1) | Fermented dairy foods (5), Sourdough (2), Pickle (3), Sour congee (1), malt production steep water (1) |
| 11 | 1 | 1 | 1 | 1 | 3 | 2 | 1 | 5 | CC1 | Inner Mongolia (1) | Sourdough (1) |
| 12 | 3 | 1 | 1 | 1 | 1 | 1 | 1 | 2 | CC1 | Inner Mongolia (1) | Sourdough (1) |
| 13 | 1 | 1 | 1 | 1 | 1 | 1 | 1 | 1 | CC1 | Inner Mongolia (1) | Sourdough (1) |
| 14 | 1 | 1 | 1 | 1 | 1 | 2 | 1 | 1 | CC1 | Inner Mongolia (1) | Sourdough (1) |
| 15 | 1 | 1 | 1 | 1 | 1 | 2 | 1 | 10 | CC1 | Inner Mongolia (1) | Sourdough (1) |
| 16 | 2 | 1 | 1 | 1 | 1 | 2 | 3 | 3 | S3 | Inner Mongolia (7) | Fermented dairy foods (7), |
| 17 | 1 | 1 | 1 | 11 | 1 | 2 | 1 | 3 | CC1 | Inner Mongolia (1) | Fermented dairy foods (1), |
| 18 | 1 | 1 | 7 | 1 | 1 | 7 | 1 | 2 | S4 | Inner Mongolia (1) | Fermented dairy foods (1) |
| 19 | 1 | 1 | 1 | 1 | 1 | 1 | 2 | 4 | CC3 | Inner Mongolia (7) | Fermented dairy foods (1) |
| 20 | 1 | 1 | 1 | 1 | 3 | 2 | 1 | 1 | CC1 | Inner Mongolia (5) | Fermented dairy foods (3), Fermented dairy foods (2), |
| 21 | 1 | 1 | 1 | 1 | 7 | 1 | 2 | 12 | S5 | Inner Mongolia (1), Tibet (1) | Fermented dairy foods (2) |
| 22 | 1 | 3 | 10 | 5 | 8 | 4 | 1 | 2 | CC10 | Inner Mongolia (1), Sichuan (1) | Fermented dairy foods (2) |
| 23 | 1 | 1 | 3 | 4 | 1 | 1 | 7 | 2 | CC5 | Inner Mongolia (7) | Fermented dairy foods (1), Sour congee (6) |
| 24 | 1 | 1 | 5 | 1 | 10 | 1 | 1 | 1 | S6 | Inner Mongolia (3), Sichuan (2) | Fermented dairy foods (3), Pickle (2) |
| 25 | 5 | 1 | 1 | 1 | 1 | 5 | 9 | 1 | S7 | Inner Mongolia (1), Sichuan (2) | Fermented dairy foods (1), Pickle (2) |
| 26 | 1 | 4 | 1 | 1 | 3 | 2 | 1 | 1 | CC1 | Inner Mongolia (3) | Fermented dairy foods (3) |
| 27 | 1 | 1 | 2 | 3 | 4 | 1 | 1 | 1 | CC4 | Dornogov, Mongolia (6), Qinghai,China (1) | Fermented dairy foods (7) |
| 28 | 1 | 1 | 1 | 6 | 11 | 2 | 3 | 1 | CC9 | Xinjiang (2) | Fermented dairy foods (2) |
| 29 | 1 | 1 | 2 | 3 | 3 | 1 | 4 | 1 | CC4 | Xinjiang (1) | Fermented dairy foods (1) |
| 30 | 1 | 1 | 1 | 6 | 1 | 2 | 3 | 1 | CC9 | Xinjiang (1) | Fermented dairy foods (2) |
| 31 | 1 | 1 | 2 | 3 | 4 | 1 | 4 | 1 | CC4 | Xinjiang (2) | Fermented dairy foods (1) |
| 32 | 1 | 1 | 2 | 3 | 3 | 1 | 1 | 1 | CC4 | Xinjiang (1) | Fermented dairy foods (1) |
| 33 | 1 | 1 | 1 | 3 | 4 | 1 | 1 | 1 | CC4 | Qinghai (3),Sichuan (1) | Fermented dairy foods (2), Fermented dairy foods (2) |
| 34 | 1 | 2 | 1 | 1 | 1 | 3 | 2 | 2 | CC2 | Inner Mongolia (1), Tibet (2) | Sour congee (1), Fermented dairy foods (2) |
| 35 | 8 | 3 | 1 | 15 | 8 | 4 | 1 | 9 | S8 | Tibet (1) | Fermented dairy foods (1) |
| 36 | 1 | 1 | 1 | 12 | 12 | 1 | 8 | 6 | S9 | Tibet (1) | Fermented dairy foods (1) |
| 37 | 1 | 1 | 1 | 1 | 1 | 3 | 2 | 2 | CC2 | Inner Mongolia (2) | Sour congee (2) |
| 38 | 1 | 1 | 4 | 2 | 1 | 1 | 1 | 2 | CC8 | Inner Mongolia (1), Sichuan (3) | Sour congee (1), Pickle (3) |
| 39 | 6 | 1 | 1 | 1 | 1 | 1 | 6 | 2 | CC7 | Inner Mongolia (1) | Sour congee (1) |
| 40 | 6 | 1 | 1 | 1 | 1 | 5 | 6 | 2 | CC7 | Inner Mongolia (1) | Sour congee (1) |
| 41 | 6 | 1 | 1 | 14 | 1 | 1 | 1 | 1 | S10 | Inner Mongolia (1) | Sour congee (1) |
| 42 | 1 | 1 | 6 | 1 | 1 | 2 | 1 | 2 | CC6 | Inner Mongolia (1) | Sour congee (1) |
| 43 | 1 | 1 | 1 | 1 | 9 | 2 | 2 | 2 | S11 | Inner Mongolia (1) | Sour congee (1) |
| 44 | 1 | 1 | 1 | 1 | 1 | 2 | 1 | 3 | CC1 | Inner Mongolia (1) | Sour congee (1) |
| 45 | 2 | 1 | 1 | 1 | 1 | 1 | 1 | 2 | CC1 | Sichuan (3) | Pickle (3) |
| 46 | 1 | 2 | 1 | 13 | 1 | 1 | 2 | 2 | CC2 | Sichuan (1) | Pickle (1) |
| 47 | 1 | 1 | 1 | 1 | 1 | 1 | 1 | 2 | CC1 | Sichuan (19) | Pickle (19) |
| 48 | 1 | 2 | 1 | 7 | 1 | 1 | 2 | 2 | CC2 | Sichuan (3) | Pickle (3) |
| 49 | 1 | 2 | 1 | 8 | 1 | 1 | 2 | 2 | CC2 | Sichuan (1) | Pickle (1) |
| 50 | 1 | 1 | 1 | 2 | 1 | 1 | 1 | 1 | CC1 | Sichuan (8) | Pickle (8) |
| 51 | 2 | 3 | 1 | 1 | 1 | 1 | 1 | 2 | CC1 | Sichuan (1) | Pickle (1) |
| 52 | 1 | 1 | 3 | 4 | 1 | 1 | 2 | 2 | CC5 | Sichuan (2) | Pickle (2) |
| 53 | 2 | 10 | 1 | 1 | 1 | 1 | 1 | 2 | CC1 | Sichuan (1) | Pickle (1) |
| 54 | 1 | 6 | 9 | 2 | 1 | 1 | 8 | 2 | S12 | Sichuan (1) | Pickle (1) |
| 55 | 1 | 2 | 1 | 1 | 1 | 1 | 2 | 8 | S13 | Sichuan (2) | Pickle (2) |
| 56 | 2 | 8 | 1 | 1 | 6 | 1 | 1 | 2 | S14 | Sichuan (1) | Pickle (1) |
| 57 | 3 | 1 | 1 | 1 | 6 | 1 | 1 | 2 | CC1 | Sichuan (5), Reference strain (1) | Pickle (5), kimchi (1) |
| 58 | 2 | 1 | 1 | 1 | 2 | 1 | 2 | 4 | CC3 | Sichuan (1) | Pickle (1) |
| 59 | 4 | 1 | 1 | 2 | 5 | 2 | 1 | 2 | S15 | Sichuan (5) | Pickle (5) |
| 60 | 1 | 10 | 1 | 1 | 5 | 1 | 1 | 2 | S16 | Sichuan (1) | Pickle (1) |
| 61 | 1 | 10 | 1 | 1 | 1 | 1 | 2 | 1 | S17 | Sichuan (1) | Pickle (1) |
| 62 | 2 | 9 | 1 | 10 | 1 | 1 | 2 | 2 | S18 | Sichuan (1) | Pickle (1) |
| 63 | 2 | 11 | 1 | 1 | 1 | 1 | 1 | 2 | CC1 | Sichuan (1) | Pickle (1) |
| 64 | 9 | 1 | 4 | 2 | 1 | 6 | 1 | 2 | S19 | Sichuan (1) | Pickle (1) |
| 65 | 1 | 2 | 1 | 8 | 1 | 3 | 2 | 2 | CC2 | Sichuan (1) | Pickle (1) |
| 66 | 1 | 9 | 1 | 1 | 1 | 1 | 1 | 2 | CC1 | Sichuan (1) | Pickle (1) |
| 67 | 1 | 5 | 4 | 2 | 1 | 1 | 1 | 2 | CC8 | Sichuan (1) | Pickle (1) |
| 68 | 1 | 5 | 1 | 1 | 1 | 1 | 2 | 7 | S20 | Sichuan (1) | Pickle (1) |
| 69 | 3 | 1 | 1 | 9 | 6 | 1 | 1 | 2 | CC1 | Sichuan (1) | Pickle (1) |
| 70 | 1 | 3 | 11 | 5 | 8 | 4 | 1 | 2 | CC10 | Sichuan (1) | Pickle (1) |
| 71 | 6 | 12 | 1 | 1 | 1 | 2 | 2 | 2 | S21 | Reference strain (1) | pickled cabbage (1) |
| 72 | 6 | 1 | 12 | 16 | 1 | 1 | 1 | 1 | S22 | Reference strain (1) | human saliva (1) |
| 73 | 1 | 13 | 1 | 17 | 1 | 1 | 2 | 2 | S23 | Reference strain (1) | health infant fecal samples (1) |

**Table S2.** Bacterial isolates used in this study

| isolates | Source of isolate | | | |
| --- | --- | --- | --- | --- |
|  | Region | source | Number | Year |
| IMAU10702, IMAU10120, IMAU10953, IMAU10125, IMAU10307, IMAU10327, IMAU10935, IMAU10942, IMAU10330, IMAU10335, IMAU11006, IMAU11014, IMAU11022, IMAU11027, IMAU11033, IMAU10386, IMAU10707, IMAU10718, IMAU10969, IMAU10971, IMAU11020, IMAU11023, IMAU11026, IMAU11029, IMAU11041, IMAU11048, IMAU11053,  IMAU10567, IMAU10578, IMAU10566, IMAU10570, IMAU10574, IMAU10580, IMAU10585, IMAU10572, IMAU10586, IMAU10591, IMAU10596, IMAU10602 | Inner Mongolia, China | Fermented dairy foods | 39 | 2009 |
| IMAU10062, IMAU10144, IMAU10160, IMAU10053 | Inner Mongolia, China | Fermented dairy foods | 4 | 2002 |
| IMAU10180, IMAU10188, IMAU10222,  IMAU10191, IMAU10196, IMAU10259,  IMAU10267, IMAU10269, IMAU10217,  IMAU10223, IMAU10224, IMAU10239,  IMAU10235, IMAU10266, IMAU10272, IMAU10273, IMAU10278 | Inner Mongolia, China | Sour dough | 17 | 2009 |
| IMAU70103, IMAU70005, IMAU70087, IMAU70088, IMAU70089, IMAU70090, IMAU70091, IMAU70094, IMAU70023, IMAU70098, IMAU70080, IMAU70092, IMAU70093, IMAU70099, IMAU70100, IMAU70101, IMAU70105 | Inner Mongolia, China | Sour congee | 17 | 2008 |
| IMAU20697, IMAU20113, IMAU20117, IMAU20118, IMAU20119, IMAU20120,  IMAU20015 | Dornogovi, Mongolia | Fermented dairy foods | 7 | 2009 |
| IMAU80101, IMAU80181, IMAU80182, IMAU80007, IMAU80030, IMAU80120, IMAU80126, IMAU80045, IMAU80148, IMAU80149, IMAU80005, IMAU80021, IMAU80145, IMAU80009, IMAU80013, IMAU80029, IMAU80066, IMAU80078, IMAU80084, IMAU80087, IMAU80088, IMAU80090, IMAU80130, IMAU80131, IMAU80132, IMAU80133, IMAU80134, IMAU80143, IMAU80170, IMAU80173, IMAU80174, IMAU80176, IMAU80188, IMAU80020, IMAU80057, IMAU80156, IMAU80023, IMAU80026, IMAU80028, IMAU80046, IMAU80142, IMAU80144, IMAU80151, IMAU80152, IMAU80153, IMAU80034, IMAU80042, IMAU80150, IMAU80052, IMAU80054, IMAU80065, IMAU80159, IMAU80071, IMAU80091, IMAU80092, IMAU80095, IMAU80178, IMAU80179, IMAU80099, IMAU80102, IMAU80106, IMAU80184, IMAU80185, IMAU80186, IMAU80103, IMAU80104, IMAU80109, IMAU80115, IMAU80125, IMAU80141, IMAU80158, IMAU80161, IMAU80162, IMAU80169, IMAU80171 | Sichuan, China | pickle | 75 | 2008 |
| IMAU80325, IMAU80297, IMAU80296 | Sichuan, China | Fermented dairy foods | 3 | 2009 |
| IMAU60042, IMAU60045, IMAU60055, IMAU60057, IMAU60170 | Tibet, China | Fermented dairy foods | 5 | 2007 |
| IMAU40116, IMAU40089, IMAU40082,  IMAU40001, IMAU40007 | Qinghai, China | Fermented dairy foods | 5 | 2005 |
| IMAU30001, IMAU30118, IMAU30043, IMAU30106, IMAU30114, IMAU30116, IMAU30162 | Xinjiang, China | Fermented dairy foods | 7 | 2004 |

**Table S3.** Information of type strains and reference strains

| strains | Genbank access number | Length (Mb) | G+C% | Source | preservation number | Country |
| --- | --- | --- | --- | --- | --- | --- |
| ATCC14917^T^ | ACGZ00000000 | 3.20 | 44.5 | pickled cabbage | ATCC14917 | USA |
| ST- III | CP002222 | 3.25 | 44.6 | kimchi | CGMCC0847 | China |
| 16 | CP006033 | 3.04 | 44.7 | malt production steep water | NCIMB 41875 | U.K. |
| P-8 | CP005942 | 3.03 | 44.8 | fermented cow milk | CGMCC6312 | China |
| ZJ316 | CP004082 | 3.24 | 44.6 | health infant fecal samples | CGMCC1.2167 | China |
| JDM1 | CP001617 | 3.20 | 44.7 | commercial lactic acid bacterium | CGMCC 1.2986 | China |
| WCFS1 | AL935263 | 3.31 | 44.5 | human saliva | NCIMB 8826 | U.K. |

Note: ATCC: American type culture collection

CGMCC: China General Microbiological Culture Collection Center

NCIMB: National Collection of Industrial and Marine Bacteria
