# Supplementary material for: Use of multilocus sequence typing to infer genetic diversity and population structure of Lactobacillus plantarum isolates from different sources
Source: BMC Microbiol. 2015 Oct 28;15:241. doi: 10.1186/s12866-015-0584-4 (PMC4625847; doi:10.1186/s12866-015-0584-4)
Supplement: Additional file 2: Figure S1. — Clonal genealogy inferred from ClonalFrame analysis of our data using a 50 % majority-rule consensus tree from three independent runs. The clades from the ClonalFrame analysis correspond to the shadowed populations identified in Fig. 2 and have therefore been coloured with the same colors as in Fig. 2. (DOC 90 kb) [file 12866_2015_584_MOESM2_ESM.doc]

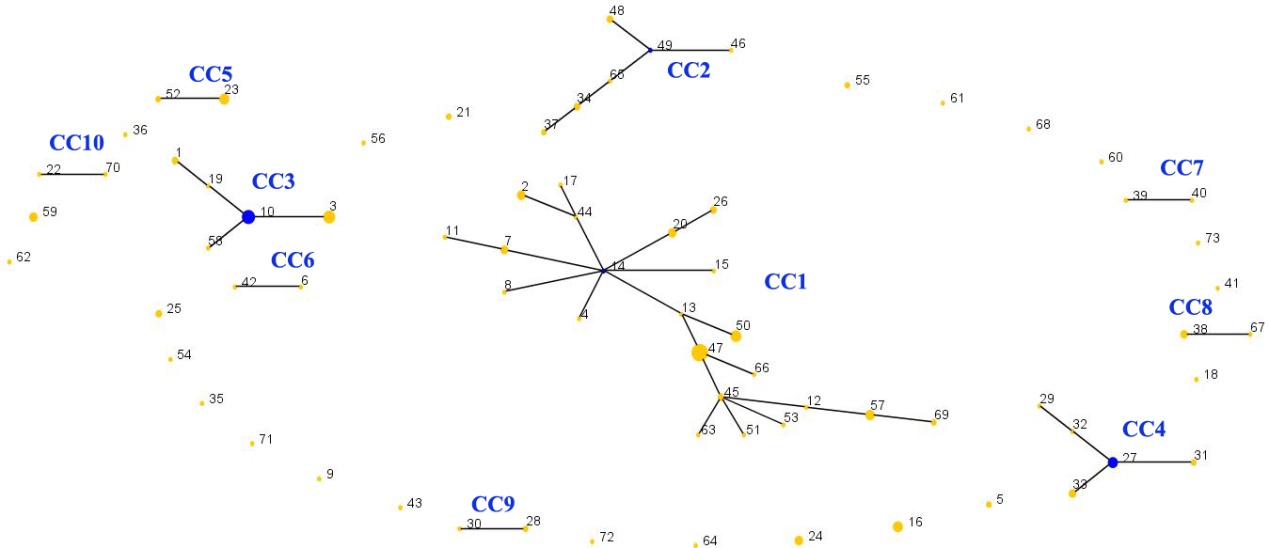


**Figure S1.** Clonal genealogy inferred from ClonalFrame analysis of our data using a 50% majority-rule consensus tree from three independent runs. The clades from the ClonalFrame analysis correspond to the shadowed populations identified in Fig. 2 and have therefore been coloured with the same colors as in Fig. 2.
